# Management of knowledge and competence through human resource information system—A structured review

**DOI:** 10.3389/fpsyg.2022.944276

**Published:** 2022-10-26

**Authors:** Khalid Rasheed Memon, Bilqees Ghani, Syed Irfan Hyder, Heesup Han, Muhammad Zada, Antonio Ariza-Montes, Marcelo Arraño-Muñoz

**Affiliations:** ^1^Graduate School of Business, Universiti Sains Malaysia, Penang, Malaysia; ^2^College of Business Management, Institute of Business Management, Karachi, Pakistan; ^3^Zia Uddin University, Karachi, Pakistan; ^4^College of Hospitality and Tourism Management, Sejong University, Seoul, South Korea; ^5^Business School, Henan University, Kaifeng, China; ^6^Social Matters Research Group, Universidad Loyola Andalucía, Córdoba, Spain; ^7^Facultad de Ciencias Sociales, Universidad Autonoma de Chile, Providencia, Chile

**Keywords:** human resource information system (HRIS), knowledge management system (KMS), HRIS expertise, top management support, strategic decision making, competency management, Iterature review, information and communication technology

## Abstract

The fourth industrial revolution will be ushered in by future high technology, and as a result, the world will face new difficulties relating to people, the environment, and profitability. Accordingly, the competitive edge and long-term viability of businesses would depend on the knowledge workers who could overcome these excruciatingly difficult obstacles and have the knowledge and competency to influence the overall performance of any type of company. But managing knowledge workers falls under the purview of human resources, and only effective human resources tools, plans, and procedures can ensure the success of this task. One such tool, which has the capacity and capability to change the whole scenario in an organization's favor, is the human resource information system (HRIS). The purpose of this structured review is to provide insight into a field of HRM (i.e., HRIS) that has largely been neglected by other reviews of the literature and has only been briefly discussed by a small number of publications published in reputable, top-tier journals. A customized HRIS framework is the result of this structured literature review for managing knowledge and competence. The study presents the content analysis of 48 articles, systematically and purposefully selected for this literature review, published during the past three decades. The study has several implications for policymakers and HR practitioners.

## Introduction

Information and communication technology (ICT) facilitates new ways of generating, searching, and distributing information and knowledge (Dewett and Jones, 2001). Knowledge management information systems are the tools that affect the management of knowledge (Ryu et al., 2003). For instance, competence analysis, development discussions, strategic orientation, training, or recruiting processes become handier when the information is stored electronically in one place (Laakso-Manninen and Viitala, 2007). Since knowledge has become a critical source for achieving sustainable competitive advantage (Markova, 2012) in hyper-competitive environments, organizations are investing millions in its management and utilization (Alavi and Leidner, 1999; Dewett and Jones, 2001). Future businesses appear to be highly reliant on knowledge workers who have the knowledge and skills to impact the whole maneuver of any corporation; they can seize the catastrophically difficult tasks relating to people, the planet, and profitability. In this context, the automation/digitalization of industrial processes based on artificial intelligence, networking of mobile devices, the internet of things, robots, cloud computing, etc., will also play a significant role in boosting the need for these knowledge workers (Memon and Ooi, 2021).

Knowledge retention strategies have emerged in response to the growing recognition of knowledge as an asset. Many people have come to believe that knowledge management strategies and practices are the keys to an effective knowledge management system. Technology advancements, especially in the field of information and communication technology (ICT), have been critical in laying the groundwork for network architecture and knowledge production; they are essential to the success of businesses today (Gedam, 2010). There has been a lot of money put into ICT solutions such as online libraries of knowledge. However, if a reservoir of knowledge lacks structure, it becomes impossible to retrieve useful knowledge from it. When put into practice, this refers to the labels that are used to classify knowledge databases. An ideal knowledge structure would faithfully reflect the knowledge's typical use (Venkitachalam and Willmott, 2015; Škrinjarić, 2022). Databases should be built with the expectation that they will undergo regular revision. This will make future modifications easier and cheaper to implement. One of the best tools for this purpose is the human resource information system (HRIS). It can be used not only for routine human resource tasks but also for the management of knowledge (Olughor, 2016). HRIS has been defined as almost similar to the knowledge management system (KMS). “It is used to acquire, store, analyze, manipulate, retrieve, and distribute pertinent knowledge and is usually inculcated as a component of an organization's strategic philosophy... and it consists of data, software, hardware, people, policies, and procedures” (Tannenbaum, 1990, p. 27). However, even in larger organizations, HRIS is not used for strategic purposes, despite how effective and efficient it is. Instead, it is used for administrative tasks such as payroll services and keeping records (Altarawneh and Al-Shqairat, 2010; Spero et al., 2011; Bamel et al., 2014; Qaisar et al., 2018). This is undesirable, given that the system is not being utilized for the purpose for which it was intended and invested. It could be considered a sunk cost (Kassim et al., 2012). So, the research suggests that a customized HRIS would not only be a standard administrative tool for managing HR activities but also an effective knowledge management system.

Knowledge management is a set of strategies for identifying, creating, and presenting the competencies and experiences of businesses and their employees. Many studies indicate that human resource management (HRM) gets added value when HRM decisions are evidence of the organizational strategy. However, useful knowledge regarding how HR influences strategic decision-making processes and contributes to being a strategic partner through the implementation of HRIS is limited. Furthermore, most of the studies have focused only on the implementation process, hurdles/factors regarding effective HRIS (Ngai and Wat, 2006; Tansley and Newell, 2007; Razali and Vrontis, 2010; Khan et al., 2017), and accomplishing competitive advantage (Browning et al., 2009), usage, benefits, and barriers (Bamel et al., 2014), HRIS adoption determinants in a limited context (Teo et al., 2007; Troshani et al., 2011; Qaisar et al., 2018), and HRIS for HR planning and development (Nagendra and Mohit Deshpande, 2014).

To the same extent, the role of knowledge and competence management has been discussed in the literature along with their concepts, importance, structures, dissemination, and management techniques (refer to for instance; Nonaka and Takeuchi, 1995; Ruggles and Holtshouse, 1999; Carlile, 2004; Dalkir, 2005), KM technologies and applications (Liao, 2003), HRM applications of knowledge-based systems (Martinsons, 1997), HRD and competency management (Schmidt and Kunzmann, 2006), and knowledge management implementation (Wong, 2005). There were a few empirical studies on organizational culture and knowledge management (Alavi et al., 2005), KM, IoT and open innovation (Santoro et al., 2018), case study on knowledge management and HR practices (Godbout, 2000), KM, HRIS, and decision support system (Zhang et al., 2012) effects of information technology on KM system (Tseng, 2008; Vahedi and Irani, 2011). Furthermore, a working paper has examined only a single part of HRIS, for instance, HR planning concerning knowledge and competence management (Haapasilta, 2010).

Since the above-cited studies have discussed all such issues very concisely and as per the scope of the study, this has resulted in scattered knowledge regarding HRIS and KM. Therefore, there is a gap and a need to describe, synthesize, and develop a holistic perspective of how HRIS can acquire, store, handle, disseminate, and resolve the issue of the management of knowledge and competencies. Such an HRIS perspective can be a valuable resource for organizations and save millions of dollars, having dual benefits and advantages. The study would open a new horizon for HR and knowledge management practitioners and researchers since KM researchers have suggested separate KM systems, whereas HRIS has been dealt with incoherently. Furthermore, less is known about HRIS's influence on strategic decision-making and allied outcomes. But these outcomes are important for business success as well as for HR professionals and management. So, this study would focus on HR's strategic role and influence on strategic decision-making through HRIS, thus developing the professional's interest in HRIS application. Also, it would highlight the importance of the usage of HRIS for the enhancement of HR's role as the organization's strategic partner. Also, the proposed study could help HR professionals see how they can use HRIS to gain a competitive edge and make their businesses successful.

Thus, this research would carry out an in-depth analysis of the published research and perform an assessment of the “Relationship of HRIS with knowledge and competency management.” Furthermore, the said study has purposefully been designed in such a way that it should describe, discuss, and evaluate all pertinent parameters appropriate to the consideration of HRIS as a tool for knowledge management. This was done, so that in-depth analysis of the published research becomes possible, and it can be carried out in the form of a narrative review for greater comprehensiveness. Accordingly, an extensive literature review would be conducted to synthesize the reasons for the underutilization of the HRIS. The study would also look at the importance, benefits, drawbacks, effects, and functions of the HRIS, as well as its role in knowledge management, its role in improving and growing human capital, and how the HRIS improves HR's role.

## Review of literature

### What is human resource information system (HRIS)?

Due to payroll activities and increasing tax regulations, technology has been used by HR since the 1940's. However, despite its quick start, the HRM function has been the last to get automated. The reasons were data intensiveness and complexity, since HRIS has been very difficult to develop and implement, as compared to accounting and supply chain systems (Kavanagh and Johnson, 2017). Therefore, it is only recently that larger organizations have implemented HR systems and started e-recruitment, performance appraisal applications, employee information management, training services, succession planning, and many more (Nankervis et al., 2021). Broadly as a whole, these systems are referred to as human resource information systems (Dery et al., 2009).

In general, HRIS is defined as “A system used to acquire, store, manipulate, analyze, retrieve, and distribute pertinent information about an organization's human resources” (Kassim et al., 2012, p. 603). In earlier days, HRIS was used as a transaction processing and management control system (Kassim et al., 2012). The three most stimulating HR functions were recruiting, payroll, and basic employee records as per the surveys (Haapasilta, 2010); however, with time, HRIS and decision support system (DSS) were formerly discussed together, in the literature. HRIS not only improves transactional processes but also helps in the design and implementation of policies and practices in accordance with organizational/business objectives, assuring the contribution of human capital (Kassim et al., 2012). Through the automation of administrative procedures and activities and the combination of enhanced access to metrics, HR's role may be expanded, allowing for a more strategic contribution (Dery et al., 2009). An HRIS is capable of spotting patterns, evaluating and controlling expenses, comparing the firm to its competitors, and generating reports. Recent HRIS advancements appear to have a considerable impact on HR strategy, integrating new technologies into the firm's goals and objectives. When an organization's enterprise resource planning (ERP) system is integrated with its human resource information system (HRIS), it gains a significant competitive edge over its rivals. According to their usage, there are two varieties of HRIS: “unsophisticated” and “sophisticated.” Payroll and benefits administration, as well as the computerized maintenance of employee absence data, are characterized as “unsophisticated.” It is considered “sophisticated” to use HRIS for recruiting and selection, HR planning, performance evaluation, training, and development (Nagendra and Mohit Deshpande, 2014).

Human resource information system's seamless, automated efficiency not only gives a manager time to develop firm-level improvement strategies, but its sophisticated reporting facilities also enable the user to generate advanced manpower reports and queries. The reports include the value of an employee's training, his service history in the organization, an employee's competencies and potential areas, employee's financial value, and so on (Argyris and Schön, 1978; Tansley and Newell, 2007; Khan et al., 2017). Managing complex entities and assessing and managing a variety of competencies is now a core requirement of the organization. Recent technologies make it possible to manage an employee's whole working life cycle, from scheduling/planning the recruitment period through retirement or termination of employment (Haapasilta, 2010). HRIS collects and keeps all the data that the manager(s) or department head(s) require for almost every HRM function and then automates nearly all ancillary operations. HRIS enables double-loop feedback on learning, which fosters organizational transformation and discussion, intra-organizational communication and decision-making, and shared visions. Thus, the system covers operations such as hiring, competency analysis, administration of continuing professional education, and mentoring (Argyris and Schön, 1978; Khan et al., 2017).

### The concepts of competence and knowledge

#### Competence

The term “competency” was first used in the field of education to describe the actions of trainee teachers. It was then used in the management field in the United States to define the qualities that employees must have to carry out their duties effectively (Škrinjarić, 2022). Competencies are not viewed as job requirements, but rather as what makes workers effective in their roles (Mitchelmore and Rowley, 2010). White (1959) coined the term “competence” to define the personal qualities that lead to successful interaction (of the individual) with one's environment (the job), which in turn leads to high performance and enthusiasm. Berio and Harzallah (2005) define competency as “a way to put into practice some knowledge, know-how, and also attitudes in a specific context” (p. 21). According to Garavan et al. (2001), intellectual capital comprises both tangible and intangible aspects. The intangible type consists of competence and relational resources, where “Competence is defined as the ability to perform a given task and is conceptualized to exist at two levels; individual in the form of knowledge, skill, and aptitude and organizational in the form of databases, technology, processes, and procedures.” “Competencies are the measurable or observable knowledge, skills, abilities and behaviors that are essential for work success” (p. 49). Employers can plan: (a) how they will organize and develop their workforce; (b) determine which job classifications best fit their business needs; (c) recruit and select the best employees; (d) effectively manage and train employees; and (e) develop staff to fill future vacancies by selecting the appropriate competencies. Knowledge competencies are defined “as a practical or theoretical grasp of a subject; skill and ability competencies are defined as natural or taught capacity to accomplish actions; and behavioral competencies are defined as patterns of action or conduct” (Schmidt and Kunzmann, 2006, p. 9).

To improve overall efficiency and cut possible expenses associated with on-the-job training, organizations (employers) utilize competence models. Through these competence models, organizations choose employees with a certain set of competencies (competence inventory) for the roles needing that particular competence inventory. In addition, for current workers, these competency criteria point to the necessity of developing an on-the-job training strategy (Husain et al., 2010). Nevertheless, competence models may be viewed by the individual (the employee) as a set of recommendations that highlight the competencies that are now necessary for the labor market. People can improve their employability and income while also cutting expenditures associated with their job hunt using this information. Thus, competence has been recognized as crucial to the success of businesses and organizations in many different fields, including but not limited to core business operations, customer relationships, financial matters, and so on (Lichtenberg et al., 2007).

The proliferation of modern organizations and, by extension, knowledge-intensive occupations can be attributed to the rising value placed on information and knowledge. Managers in the information technology (IT), banking and finance, insurance business, and service management industries of today use a different set of competencies than their predecessors did in the same roles, three decades ago (Nikitina and Lapina, 2019). Many scholars over the past few decades (Liu, 2006; Ingason and Jónasson, 2009; Wiek et al., 2011; Derwik et al., 2016) have pointed to managers' communication, negotiating, and relationship-building skills as essential “soft skills.” Previous research found that the labor market requires more complex transferable social skills (Kuokkanen et al., 2013). This finding suggests that skills and competencies based on personality traits are even more in demand.

Managers in the modern era are tasked with making decisions and managing innovations based on the data. They must have a solid foundation of technical knowledge and competence in the field (Nikitina and Lapina, 2019). This allows them to exert leadership skills over the business, motivate the most cutting-edge employees, and serve as a driving force behind technological advancements. Expertise in the business sector and an emphasis on the needs of customers and other stakeholders have been cited as keys to success in today's knowledge-based economy (Nikitina and Lapina, 2019).

Industry 4.0 organization is not achievable without the necessity for information transparency. This is made impossible without all these technological advancements and the extraordinarily fast dissemination of inventions that is 10 times faster than it was 150 years ago (Memon and Ooi, 2022). The emergence of new forms of competencies, knowledge production, and dissemination *via* online communities of practice and social media provides supporting evidence for this claim (Sarka and Ipsen, 2017). Therefore, it is recommended that the company takes preventative measures to secure its interests by coordinating the knowledge, abilities, and tools of its human resources department. Organizations all over the world carry out knowledge management (KM) procedures to evaluate the knowledge and competence of their employees and to disseminate and develop new knowledge (Rosha and Lace, 2016; Zieba et al., 2017).

#### Knowledge

The Concise Oxford Dictionary defines knowledge as “awareness or familiarity gained by experience (of a person, fact, or thing).” This definition adheres to the tenets held by the empiricist school of philosophy, which is often known as the scientific method (Biggam, 2001). They believe that first-hand experience is the only way to acquire knowledge. Experience is indeed the best way to learn new things, but it does not mean there are no other paths to enlightenment. It is also possible to arrive at this state by logical deliberation (Wahono and Chang, 2019a). To emphasize, for anything to be considered knowledge, it must be true (this helps distinguish between fact and belief). This latter point could be helpful in one fundamental aspect of the development of the university website. This puts emphasis that those responsible for their university website must be certain, before displaying information. Furthermore, what is presented is accurate and is not simply based on the beliefs. This latter point could help in this regard (Wahono and Chang, 2019b).

Contemporary authors differentiate among data, information, and knowledge. Data are defined as “raw facts or simple comments regarding the status of the world;” information is defined as “data in some context, or with a little human interpretation applied;” whereas “knowledge is information along with direction for action, i.e., knowing how to proceed with given information” (Liew, 2007).

Broadly, knowledge could be grouped into individual knowledge and organizational knowledge. “Knowledge that resides in an individual's mind is individual knowledge, whereas organizational knowledge is developed through interaction between people, technologies, and techniques” (Yahya and Goh, 2002, p. 458). Tacit and explicit knowledge are two further classifications of organizational knowledge. “Tacit knowledge is something which is contained in a person's head and is hard to express, put in writing, and codify” (Nonaka and Takeuchi, 1995). Examples can be how to secure a deal with a meticulous type of customer or how to design and develop an attractive advertising campaign. Tacit knowledge is of immense importance to organizations because it contains knowledge that leads to valuable strategies, guiding principles, practices, and measures. On the other hand, explicit knowledge is “that which can be easily expressed, written down, codified, and shared” (Nonaka and Takeuchi, 1995). Instructions and standard operating procedures on how to bake pastry are the illustrations of explicit knowledge.

The author, Hislop (2013), also considers tacit knowledge to be the most important type of knowledge for a business. While Davenport and Prusak (1998) agree that knowledge sharing is crucial to a company's success, they argue that it is also one of the most difficult things to define and quantify. This is something that develops and is used in the heads of the knowledgeable. The phrase “it becomes ingrained in organizational routines, procedures, practices, and norms” refers to the phenomenon whereby knowledge spreads across an organization and eventually becomes part of the fabric of the company (p. 24). In a similar disposition, Brown and Duguid (2001) point out that Polanyi (1996) concludes “that knowledge always has an inarticulate component” and that he “was not, then, arguing for two forms of knowledge, but for two dimensions—two interdependent dimensions” (p. 203–204). To rephrase, Polanyi's explicit dimension of knowledge is found on the foundation of tacit knowledge that has been “interiorized” in the past. According to Botha et al. (2014), however, the distinction between these theoretical opposites is vital. It is important to note that these two types of knowledge reflect a spectrum rather than independent categories of knowledge (when trying to investigate knowledge creation and management). The widespread codification and personalization techniques provide a good example of the mobilization of each form of knowledge in business (Hansen et al., 1999; Ajith Kumar and Ganesh, 2011). “The codification strategy is concerned with extracting explicit knowledge from the person who developed it, storing it in databases, and promoting its subsequent reuse by anyone who needs it”, as stated by Ajith Kumar and Ganesh (2011), “while the primary concern of the personalization strategy is tacit knowledge and its transfer among people, enabled by facilitating direct interactions between people, by connecting people with each other” (p. 119). Firms that have to constantly innovate and deliver customized solutions in a short amount of time and that need to protect their knowledge from the competition will benefit from personalization (Bosua and Venkitachalam, 2013). However, some of the drawbacks of personalization include knowledge hoarding by individuals who are afraid of losing their competitive advantage and key employee poaching by competitors who want to acquire critical tacit knowledge (Mukherji, 2005; Ajith Kumar and Ganesh, 2011).

### Management of knowledge and competencies

Knowledge management has attracted a great deal of attention from academics and professionals in a wide range of industries because of its importance to the survival and development of enterprises (Rehman et al., 2021). In the broader sense, knowledge management (KM) is the subject that formalizes methods and techniques for understanding, leveraging, and utilizing knowledge resources at the organizational level. KM helps the organization compete since KM is supposed to enhance innovativeness and responsiveness (Keshtidar et al., 2017; Santoro et al., 2018; Bootz et al., 2019). The KM process involves “the design, review, and implementation of both social and technological processes to improve the application of knowledge in the collective interest of stakeholders,” whereas IT facilitates successful KM (Donate and Guadamillas, 2015; Nieves et al., 2016).

Knowledge management may be considered as a method of storing/recording the knowledge kept by various organizational departments. This ensures the accumulation of that knowledge for the rest of the organization. At least one should consider the four crucial processes of creation. These are storage/retrieval, transfer, and knowledge application along with communication networks for the transfer of knowledge horizontally and vertically, as desired (Courtney, 2001).

**Figure 1:**
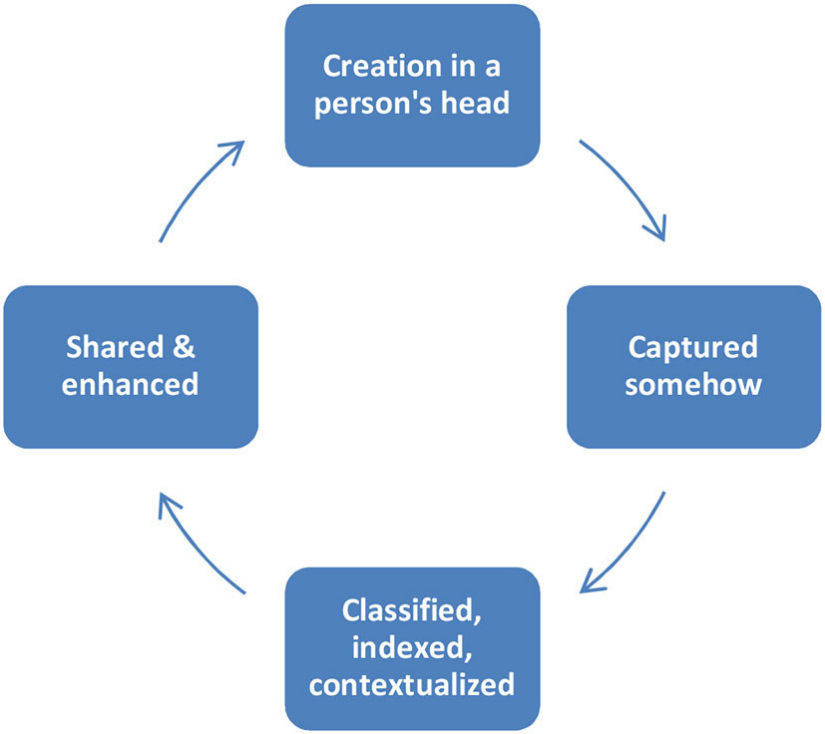
Bock's knowledge creation cycle (Bock et al., 2005).

Bock et al. (2005) also defines KM as a four-part process that consists of a loop (refer to Figure 1). Knowledge is created in a person's head. It is captured somehow. It is placed on a document in a report, put into a computer system, or some kind of repository, or simply memorized. Knowledge is classified and customized. The classification is indexed or can comprise the adding up of keywords. Customization can add a framework, background, or many other things to make it easier to reuse whenever needed at later stages. The analysis of this stage is how simply people in the firm will be proficient at locating and utilizing the knowledge when they require it. While knowledge is shared and utilized, it is customized by the people who use it. This brings us back to knowledge creation (Courtney, 2001).

A knowledge management system makes efficient use of tangible assets as its primary goal is the collection and analysis of knowledge to enhance performance and streamline process management. Because of this, intelligent infrastructures and interaction-based collaborative methods can help corporate innovation processes by building a cognitive architecture that can make sure the knowledge is used and kept. This means that KMS has an impact on the company's success since it fosters innovation, which in turn strengthens the company's position in the market (Vaio et al., 2021).

On the other hand, “competence management is how organizations manage the competencies of the corporation, the groups, and the individuals” (Berio and Harzallah, 2005). The main purpose is to define and maintain these competencies continuously as per the firm's objectives. A competency is, say, some kind of way to put into practice skills or know-how and attitudes within a particular context. Competence management has been recognized as exceptionally essential for the accomplishment of a firm's goals, complementary to, for example, core business processes, financial issues, customer relationships, and so on. Competence management can be ordered into four kinds of processes (i.e., may include several sub-processes):

- Competence identification: This includes the process of how and when to identify as well as define competencies required (i.e., now or in the future) to carry out tasks, missions, and strategies.
- Competence assessment: This refers to the relationship between individual and required competencies, i.e., (i) when and how to identify and define competence acquired by individuals and/or (ii) when and how an organization can decide that a worker has acquired particular competencies.
- Competence acquisition: This refers to an organization's decision regarding how to acquire some competencies in an intended way and when.
- Competence usage: This includes how to utilize the knowledge or knowledge regarding the competencies developed and transformed through identification, assessment, and acquisition processes; for example, how to recognize gaps among required and acquired competencies, and some workers still need to attend required training, how to find key employees having key competencies and so on.

Knowledge management system makes it possible to record the expertise of specific people and share that knowledge with the rest of the organization. Furthermore, a knowledge management system (KMS) may be seen as an enabler of knowledge management from the point of view of the knowledge-based approach. An efficient KMS will typically consist of the following three components (Santoro et al., 2018):

- Information technology infrastructures are also known as physical technology assists in the efficient management of knowledge and include things such as hardware, software components, extranet, intranet, and local area networks (Soto-Acosta and MeroñO-Cerdan, 2008).
- Technologies facilitate collaboration, such as online message boards, shared databases, document archives, and workflow systems (Merono-Cerdan et al., 2007).
- The adoption of ICT is capable of integrating a variety of collaborative technologies and whose usage orientation is inclined toward achieving three key implementation goals (Lopez-Nicolas and Soto-Acosta, 2010): (a) the ICT informative orientation seeks to provide commercial information to a variety of stakeholders, regardless of organizational or functional boundaries; (b) the ICT communicative orientation makes it possible to reduce costs and interact with a variety of business agents both inside and outside the organization; and (c) the ICT workflow orientation establishes electronic processes within corporate technologies (Santoro et al., 2018).

## Methodology

### Research design

The findings of this study are based on credible previous research. The Web of Science, Scopus, Emerald, Google Scholar, and Science Direct databases were used for this investigation in February 2021. This research methodology is based on four steps, comprised of data identification, initial data screening, data eligibility determination, and finally, data inclusion, as shown in Figure 1. A blend of specific terms was used in the research study, taken from the title and abstract, and keywords such as “Human resource management system, human resource information system (HRIS), and knowledge management system” have been used (refer to Table 1). All references published between 1990 and 2021 are now included in the publication year as a filter since the work on the human resource management system started in the 90's. To begin, a list of all the research was compiled that had been finalized and published. Second, the articles that did not meet the qualifying criteria were eliminated, such as the articles' titles, relevancy, or content. After that, articles were narrowed down to a final 80 pieces by reading the whole thing a third time.

**Table 1:** Initial search results and a number of papers appeared.

**Keywords**	**Results (no. of articles)**	**Limit to**
Human resource management system	37,434	Article title, keywords, abstract
Human resource information system and knowledge management system	2,153	//
Human resource information system	26,014	//
HRIS	278	//
Knowledge management system	63,851	//
Competency management and human resource information system	241	//
Total	129,971	–

### Data search and screening

First, 129,971 papers were derived using the combinations of keywords. The initial search results of the Scopus database are presented in Table 1. Initially, the researchers retrieved the data from various sources, including conference papers, books, book chapters, and articles. Afterward, the researcher mainly focused on the articles published in the English language published between the period 1990 and 2021. Then, the research was limited to the subject area of “Business management and accounting,” which brought the articles to a total of 15,408 (refer Table 2).

**Table 2:** Screening of articles.

**Keywords**	**Results (no. of articles)**	**Limit to subject area**
Human resource management system	4,070	Business management and accounting
Human resource information system and knowledge management system	279	//
Human resource information system	1,457	//
HRIS	99	//
Knowledge management system	9,449	//
Competency management and human resource information system	54	//
Total	15,408	–

Since the number of articles for review (15,408) was still large, they were further screened. For instance, the articles on the “knowledge management system” keyword (9,449) were further screened through the use of some other sub-keywords; for instance, articles with keywords “knowledge management system, information management, administrative data processing, managers, data mining, human, management information system, human resource management” were used and it brought 4,202 articles in this category.

The category of articles included in the keyword “human resource management” (4,070) was further decreased by limiting articles to the sub-keywords of “knowledge management, human resource management, information management, technology, personnel management, technology, and management information systems” (1,447 articles). Thus, at this screening stage, there were a total of 6,081 articles that included articles of all keywords.

**Figure 2:**
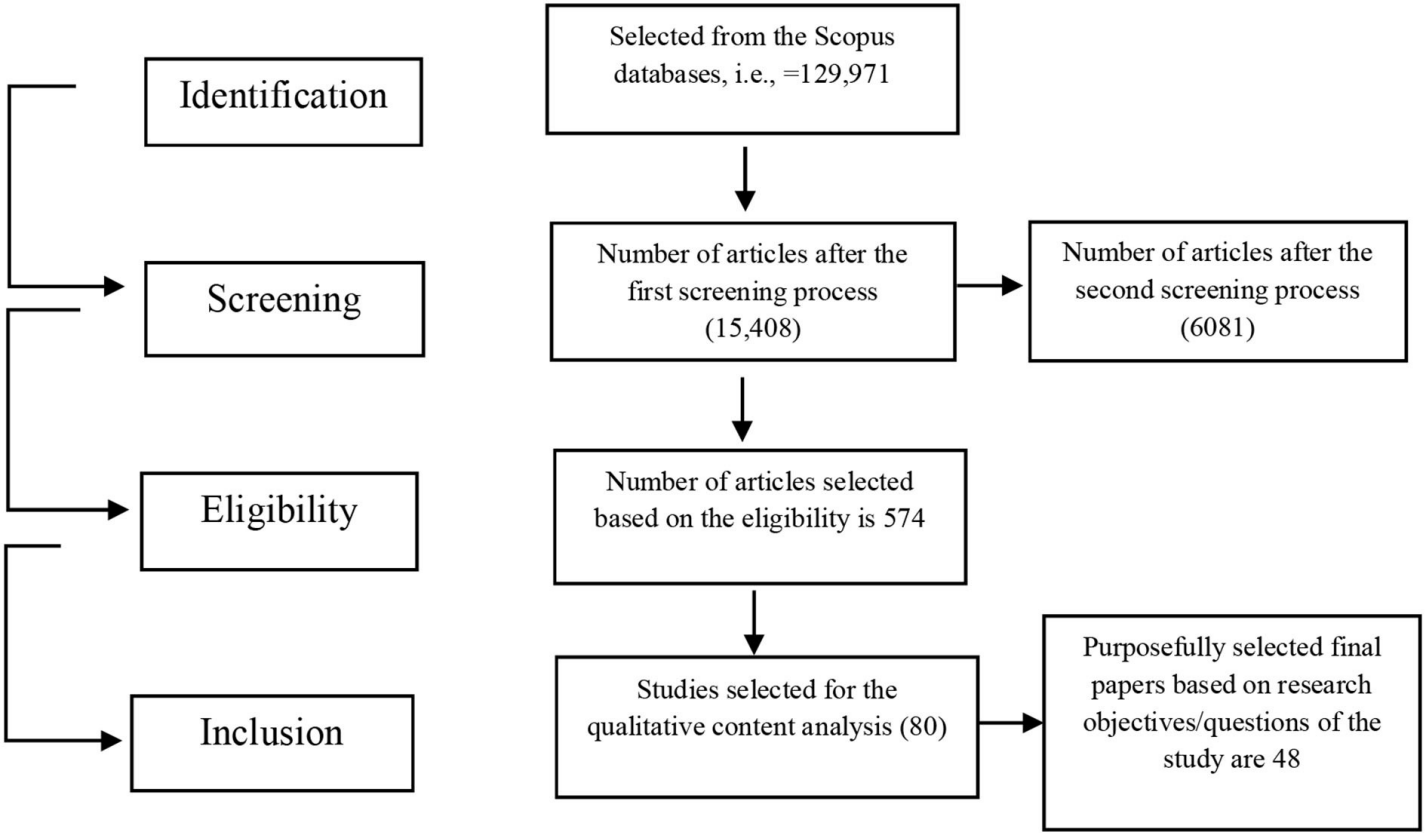
Literature search process represented through the data flow diagram.

The research then applied the third step based on inclusion/exclusion criteria as detailed in Section Exclusion/inclusion criteria. At this stage, strict criteria were applied to select the articles; those not related to the KMS and HRIS were deleted from the study's scope. Some papers were duplicates, so they were deleted, whereas some articles were not accessible in the researchers' work settings. Thus, the research study was limited to 574 articles for abstract reading since limited work has been done on HRIS and KMS. The authors read the abstracts of all these articles and still found very limited relevance to HRIS and KMS. Thus, 80 articles were selected for content analysis. These 80 articles were read completely for the qualitative review of the research questions and objectives of the study, whereby only 48 of them were finally considered suitable for the study and met the objectives/research questions (refer to Section Research question design). The sequential steps followed for the literature search & screening have been presented through flow-chart in Figure 2.

### Exclusion/inclusion criteria

#### Inclusion criteria

- Articles published in recognized and peer-reviewed journals listed in Scopus.
- Articles in English language only.
- Publication period between 1990 and 2021.
- Only journal articles.
- Articles related to the area of business management and accounting.

#### Exclusion criteria

- Articles published in non-peer-reviewed journals, magazines, thesis, reports, etc.
- Papers published before 1990.
- Papers published in languages other than English.
- Duplicated work/papers.
- Irrelevant papers/not discussing HRIS and knowledge and competency management.

### Research question design

The review is focused on a substantial proof study of HRIS activities (and related mechanisms) that enhance knowledge management and competence activities in business contexts, particularly in larger businesses.

The following review questions are considered during the analysis of the studies:

1. To explore and evaluate critically the relationship between HRIS and KMS?
2. To explore and investigate, if customized HRIS can act as and replace KMS?
3. To propose a model presenting customized HRIS as KMS.
4. To investigate the reasons for the under-utilization of HRIS including barriers?

### Research studies finally selected for literature review

The literature contains several articles on knowledge management, information systems, competency management, and HRIS. All these are different areas and they each discuss their relevant concerns and issues. However, because of unique and novel research questions, very few studies were found to fulfill the study's exact criteria, research questions, and purpose. Thus, after a thorough and rigorous review of the literature, the following studies were selected for the review process (refer to Table 3).

**Table 3:** Description of studies included in the review process.

**Sr#**	**Authors**	**Study title**	**Publication journal**	**Year of publication**	**Citations**
1	Tannenbaum, S. I.	Human resource information systems: user group implications	Journal of Systems Management	1990	195
2	Dunivan, L.	Implementing a user-driven human resource information system	Journal of Systems Management	1991	30
3	Ulrich, D., Brockbank, W., Yeung, A. K., and Lake, D. G.	Human resource competencies: an empirical assessment	Human Resource Management Journal	1995	605
4	Blackler, F.	Knowledge, knowledge work and organizations: an overview and interpretation	Organization Studies	1995	4,200
5	Kamoche, K.	Strategic human resource management within a resource-capability view of the firm	Journal of Management Studies	1996	630
6	Haines, V. Y., and Petit, A.	Conditions for successful human resource information systems	Human Resource Management	1997	257
7	Elliot, R. H., and Tevavichulada, S.	Computer literacy and human resource management: a public/private sector comparison	Public Personnel Management	1999	93
8	Alavi, M., and Leidner, D. E.	Knowledge management systems: issues, challenges, and benefits	Communications of the Association for Information Systems	1999	2,671
9	Godbout, A. J.	Managing core competencies: the impact of knowledge management on human resources practices in leading-edge organizations.	Knowledge and Process Management	2000	74
10	Brynjolfsson, E., and Hitt, L. M.	Beyond computation: information technology, organizational transformation and business performance	Journal of Economic Perspectives	2000	4,193
11	Ball, K. S.	The use of human resource information systems: a survey	Personnel Review	2001	460
12	Alavi, M., and Leidner, D. E.	Review: knowledge management and knowledge management systems: conceptual foundations and research issues	MIS Quarterly	2001	15,500
13	Courtney, J. F.	Decision making and knowledge management in inquiring organizations: toward a new decision-making paradigm for DSS	Decision Support Systems	2001	800
14	Yahya, S., and Goh, W. K.	Managing human resources toward achieving knowledge management	Journal of knowledge management	2002	880
15	Beckers, A. M., and Bsat, M. Z.	A DSS classification model for research in human resource information systems	Information Systems Management	2002	200
16	Kovach, K. A., Hughes, A. A., Fagan, P., and Maggitti, P.	Administrative and strategic advantages of HRIS	Employment Relations Today	2002	280
17	Hendrickson, A. R.	Human resource information systems: backbone technology of contemporary human resources	Journal of Labor Research	2003	429
18	Gardner, S. D., Lepak, D. P., and Bartol, K. M.	Virtual HR: the impact of information technology on the human resource professional	Journal of Vocational Behavior	2003	268
19	Mayfield, J., Mayfield, M., and Lunce, S.	Human resource information systems: a review and model development	Advances in Competitiveness Research	2003	116
20	Hempel, P.S.	Preparing the HR profession for technology and information work	Human Resource Management	2004	113
21	Carlile, P. R.	Transferring, translating, and transforming: an integrative framework for managing knowledge across boundaries	Organization Science	2004	3,651
22	Bock, G. W., Zmud, R. W., Kim, Y. G., and Lee, J. N.	Behavioral intention formation in knowledge sharing: examining the roles of extrinsic motivators, social-psychological forces, and organizational climate.	MIS quarterly	2005	5,900
23	Ngai, E., and Wat, F.	Human resource information systems: a review and empirical analysis	Personnel Review	2006	304
24	Teo, T. S. H., Lim, G. S., and Fedric, S. A.	The adoption and diffusion of human resources information systems in Singapore	Asia Pacific Journal of Human Resources.	2007	181
25	Tansley, C., and Newell, S.	A knowledge-based view of agenda-formation in the development of human resource information systems	Management learning	2007	48
26	Nusair, K., and Parsa, H. G.	Critical factors in implementing HRIS in restaurant chains	Advances in Hospitality and Leisure	2007	12
27	Tseng, S. M.	The effects of information technology on knowledge management systems	Expert systems with applications	2008	228
28	Haines III, V. Y., and Lafleur, G.	Information technology usage and human resource roles and effectiveness	Human Resource Management	2008	151
29	Ulrich, D., Younger, J., and Brockbank, W.	The twenty-first century HR organization	Human Resource Management	2008	277
30	Bondarouk, T., and Ruël, H.	HRM systems for successful information technology implementation: evidence from three case studies	European Management Journal	2008	64
31	Browning, V., Edgar, F., Gray, B., and Garrett, T.	Realizing competitive advantage through HRM in New Zealand service industries	The Service Industries Journal	2009	100
32	Delorme, M., and Arcand, M.	HRIS implementation and deployment: a conceptual framework of the new roles, responsibilities and competences for HR professionals	Int. J. Business Information Systems	2010	47
33	Razali, M. Z., and Vrontis, D.	The reactions of employees toward the implementation of human resources information systems (HRIS) as a planned change program: a case study in Malaysia	Journal of Transnational Management	2010	39
34	Spero, J. C., McQuide, P. A., and Matte, R.	Tracking and monitoring the health workforce: a new human resources information system (HRIS) in Uganda	Human Resources for Health	2011	50
35	Schalk, R., Timmerman, V., and Heuvel, S. V. D.	How strategic considerations influence decision making on e-HRM applications	Human Resource Management Review	2012	100
36	Strohmeier, S., and Kabst, R.	Evaluating major human resource information systems design characteristics – an empirical study	Int. J. Business Information Systems	2012	8
37	Kassim, N. M. Ramayah, T., and Kurnia, S.	Antecedents and outcomes of human resource information system (HRIS) use	International Journal of Productivity and Performance Management	2012	96
38	Venkitachalam, K., and Busch, P.	Tacit knowledge: review and possible research directions	Journal of knowledge management	2012	271
39	Chakraborty, A. R., and Mansoor, N. N. A.	Adoption of human resource information system: a theoretical analysis	Procedia - Social and Behavioral Sciences	2013	73
40	Bosua, R., and Venkitachalam	Aligning strategies and processes in knowledge management: a framework	Journal of Knowledge Management	2013	166
41	Bamel, N., Kumar Bamel, U., Sahay, V., and Thite, M.	Usage, benefits and barriers of human resource information system in universities	VINE: The journal of information and knowledge management systems	2014	44
42	Venkitachalam, K., and Willmott, H.	Factors shaping organizational dynamics in strategic knowledge management	Knowledge Management Research and Practice	2015	46
43	Al-Dmour, R. H., Love, S., and Al-Debei, M. M.	Factors influencing the organizational adoption of human resource information systems: a conceptual model	Int. J. Business Innovation and Research	2016	11
44	Olughor, R. J.	The relationship between human resource information system and human resource management	International Journal of Economics and Management	2016	83
45	Alam, M. G. R., Masum, A. K. M., Beh, L-S., and Hong, C. S.	Critical factors influencing decision to adopt human resource information system (HRIS) in hospitals	PLoS ONE	2016	90
46	Khan, H., Hussainy, S. K., Khan, K., and Khan, A.	The applications, advantages and challenges in the implementation of HRIS in Pakistani perspective	VINE Journal of Information and Knowledge Management Systems	2017	13
47	Keshtidar, M., Esmaeilzade Ghandehari, M. R., and Harati, M.	The effect of knowledge management through human resources information systems on customer relationship management in aquatic sport centers	Annals of Applied Sport Science	2017	4
48	Sarka, P., and Ipsen, C.	Knowledge sharing *via* social media in software development: a systematic literature review	Knowledge Management Research and Practice	2017	50

## Research insights

**Figure 3:**
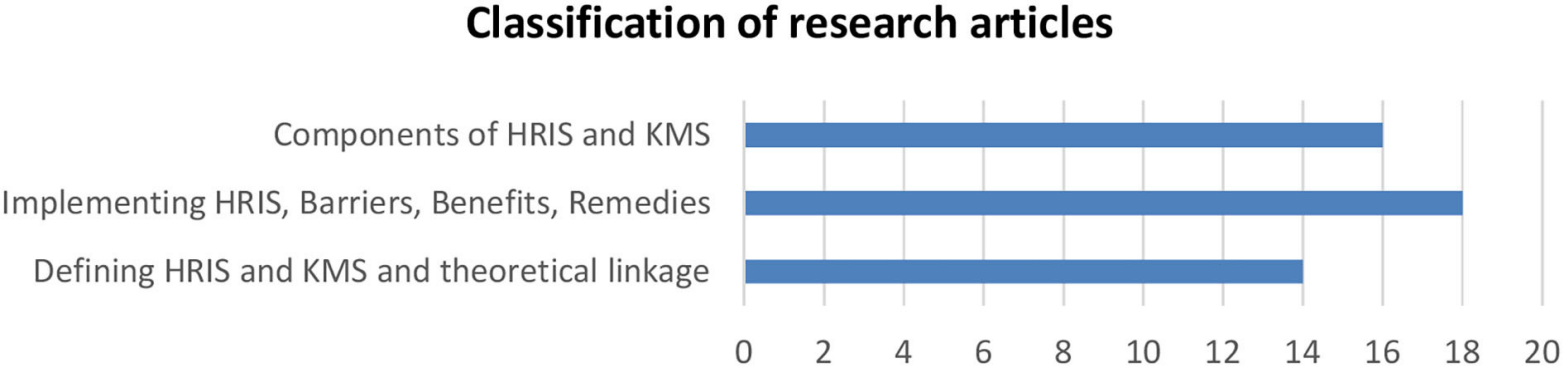
Classification of articles' dimensions.

This section presents the findings from 48 papers selected from the Scopus and ISI Web of Science databases. To suggest HRIS as KMS, the whole HRIS and KMS literature has been divided into three groups. The first group of research studies classifies HRIS and KMS studies at a higher level, delving into the concepts of HRIS, knowledge management, and competency management. There are 14 papers in this category group, accounting for about 29% of the total number of articles evaluated. The extant literature on integrating HRIS philosophy at the company level and its execution is organized in the second primary category. This is related to the impact of implementing HRIS at the firm level and supporting the HR department. It also includes the barriers, benefits, and remedies. This category includes empirical papers since the benefits and barriers of implementing HRIS have been measured. There are 18 articles in this category, accounting for 37.5% of the research considered. The third category of literature includes the constituents of literature having details regarding HRIS and KMS components. Tacit knowledge, explicit knowledge, data and information, knowledge conversion cycle, expert systems, and DSS were all significant themes in these papers. There are 16 of them, accounting for over 33.5% of all articles submitted. Figure 3 shows the individual articles utilized for each of these dimensions. However, where a study applied to several categories, the most relevant category/dimension was picked.

### Knowledge-based and resource-based views and HRM

The knowledge-based view of the firm has made a distinguishing contribution to the strategic human resource literature (Narasimha, 2000). The knowledge-based view goes beyond what the resource-based view (RBV) scholars simply view HR systems as resources (Barney, 1991). The RBV offers only a stagnant representation of the “process by which core competencies are built or lost.” It does not consider the significance of the continuous development of the competencies or their reconfiguration. The resolution of these boundaries is inculcated in an approach, based on “dynamic capabilities” [as explained by Wright et al., 2001]. The dynamic capability approach goes beyond the simple use of existing resources and is concerned with the analysis of their revitalization and development (Canzano and Grimaldi, 2012).

To gain competitive advantage, the knowledge-based view particularly focuses on employees' competencies and capabilities to leverage their knowledge and continuous education through knowledge acquisition, incorporation, and transfer (Kulvisaechana, 2005). Wright et al. (2001) state that “knowledge can be viewed as something that characterizes individuals (i.e., human capital), but it can also be shared within groups or networks (i.e., social capital) or institutionalized within organizational processes and databases (organizational capital)” (p. 714). Furthermore, Kamoche (1996) posits that “human resources are not highly valued if they fail to acquire and utilize their knowledge competencies in the workplace.” Skillful, motivated, and well-heeled human resources are the most important resources of an organization. Professional excellence (competence) has to be a key part of the organization's strategy for it to succeed.

Human resource management is all about managing, organizing and developing, applying, and assessing procedures, techniques, and programs associated with an individual in the organization (Miner and Crane, 1995; Yahya and Goh, 2002). Laakso-Manninen and Viitala (2007) explain that strategic HRM puts its efforts into finding an answer to the problem: how does an organization plan, organize, control, assess, and develop human resources to accomplish its strategy and maintain its competitive position. Thus, a human resource development (HRD) system should always incorporate personal dimensions, the organization's core competencies, and the knowledge base. A competence-based strategy sets the formation and path for HRD (Alavi and Leidner, 1999; Laakso-Manninen and Viitala, 2007).

### Proposed framework model

**Figure 4:**
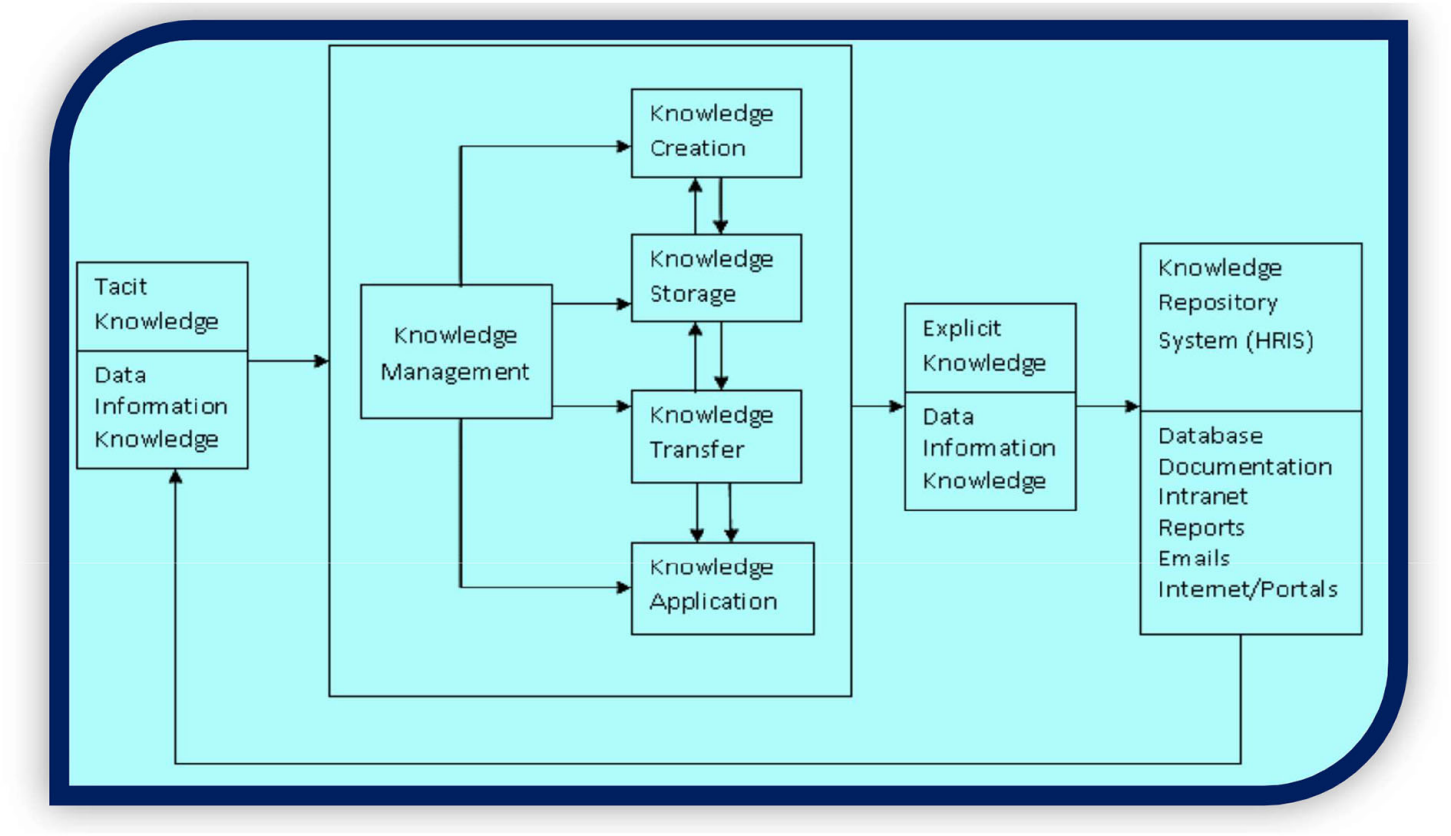
Knowledge repository system (HRIS; source: authors, based on Alavi and Leidner, 1999; Laakso-Manninen and Viitala, 2007; Gedam, 2010).

The research proposes the following model of the HRIS system (refer to Figure 4), which may be used as a customized KMS. Its various parts will be discussed sequentially in each of the following sections, which are linked with the definitions of HRIS and KMS as defined in the above section.

### Managing knowledge and competence through HRIS

Knowledge and competence have become inevitably critical, and organizations cannot succeed without proper management, utilization, and continuous improvement. In particular, in today's highly competitive era of technology and increased competition, knowledge and competence are the distinguishing features of organizations. These can become the basis for attaining competitive advantage and may influence the overall performance of the organization and business (Garavan et al., 2002; Bosua and Venkitachalam, 2013). Human capital, being described as having “possession” of knowledge and skills, is the main cause of gaining competitive advantage and thus influences the overall performance of any kind of business (Memon, 2014).

Businesses such as engineering and design services, high fashion, computer software engineering, and design, health care, management consulting, and financial services may be considered as knowledge businesses (Lei et al., 1999). These businesses are dependent on the transformation of their human capital's KSAs (knowledge, skills, and abilities) into intellectual capital (product and service offerings in the marketplace). Here, making a fortune is more reliant on the application of specialist knowledge and the management of organizational competencies than having control of resources (Blackler, 1995). In a knowledge-based society, the mismanagement of tangible resources is no longer the reason for the collapse. So, now, the focus should be diverted to the management of the intangibles, i.e., intellectual capital (Boxall et al., 2007). The transformation of human capital into intellectual capital has been very much dependent on the environment for knowledge creation and sustenance (Bootz et al., 2019). Knowledge workers intermingle with other knowledge workers to generate a knowledge-intensive product. Knowledge workers not only utilize their knowledge but also use the knowledge of co-workers as shared through information systems and artifacts (Boxall et al., 2007). So, it is very important to take care of the knowledge environment to make sure that knowledge tasks can be done.

The sophisticated information processing potentials (formatting, sorting and summarizing) in management information systems (MIS), expert information systems (EIS), and decision support systems (DSS) can be used to accomplish the transformation process and increase the worth of information by growing its form, place and time utilities (Tseng, 2008; Gedam, 2010). Apart from the conventional functions of transforming data, information systems (IS) have currently been utilized to dig out information and knowledge from accessible databases (refer Figure 3). Accordingly, expert systems (ES), case-based reasoning systems, and neural networks are all being used more and more frequently to generate new insights and identify important relationships that could provide a competitive advantage (Beckers and Bsat, 2002). IS may be applied to smoothen the progress of the gathering, accumulating, and developing of important information and knowledge. IS can help by playing a vital role in serving firms to build and activate their distinguishing knowledge and capabilities to achieve competitive advantage (Alavi and Leidner, 2001; Bondarouk and Ruël, 2008). Now, expert systems (a type of knowledge-based system) are being more widely used in businesses as a way to store, amass, and transfer the scarce but invaluable knowledge and abilities of their human resources. Some firms are also using intranets to preserve important organizational documents and discussions. Some intranets retain a record of their human resources besides their field of specialization. This helps to assist in the exploration, access, and recovery of soft knowledge and utilize search engines of that software to categorize knowledge or possessors of desired knowledge (Gedam, 2010).

### The link between human resources, knowledge and competence management

The resource-based perspective of an organization depicts an organization as a collection of competencies, knowledge accumulation, and experience (Barney, 1991). However, a knowledge-based perspective contends that skill development and continual learning can help an organization maintain its competitiveness and sustainability, whereas people are an organization's major source of sustainability (Dyer, 2004). Organizations build their human resources as a source of competitive advantage through a series of activities. These include searching for, selecting, and employing competent and dynamic employees; investing in their development for the organization's specific responsibilities; and then deploying them for the organization's gain and use (Carlile, 2004; Memon, 2014; Bootz et al., 2019; Memon and Ghani, 2021). Dyer (2004) quotes Klein et al. (1978) and Mahoney and Pandian (1992) that “human capital can be inimitable if they are trained firm-specifically as your rivals can't use them in the same way.”

Simply said, people run machines, not machines run people; hence, investment in human resources yields productivity (Becker, 1994). It is the people who will generate ideas, approaches, and tactics to deal with worst-case scenarios (Memon, 2014). According to Marshall (1890), “the most valuable of all capital is that invested in human beings” (Nerdrum and Erikson, 2001). This brings us to the function of strategic human resource development (SHRD) because human development is HRD's primary job in an organization. HRD specialists are now tasked with advising firms on future-focused strategic planning projects to create estimations on the composition, quantity, and skill level of personnel (Liu et al., 2006; Browning et al., 2009).

**Figure 5:**
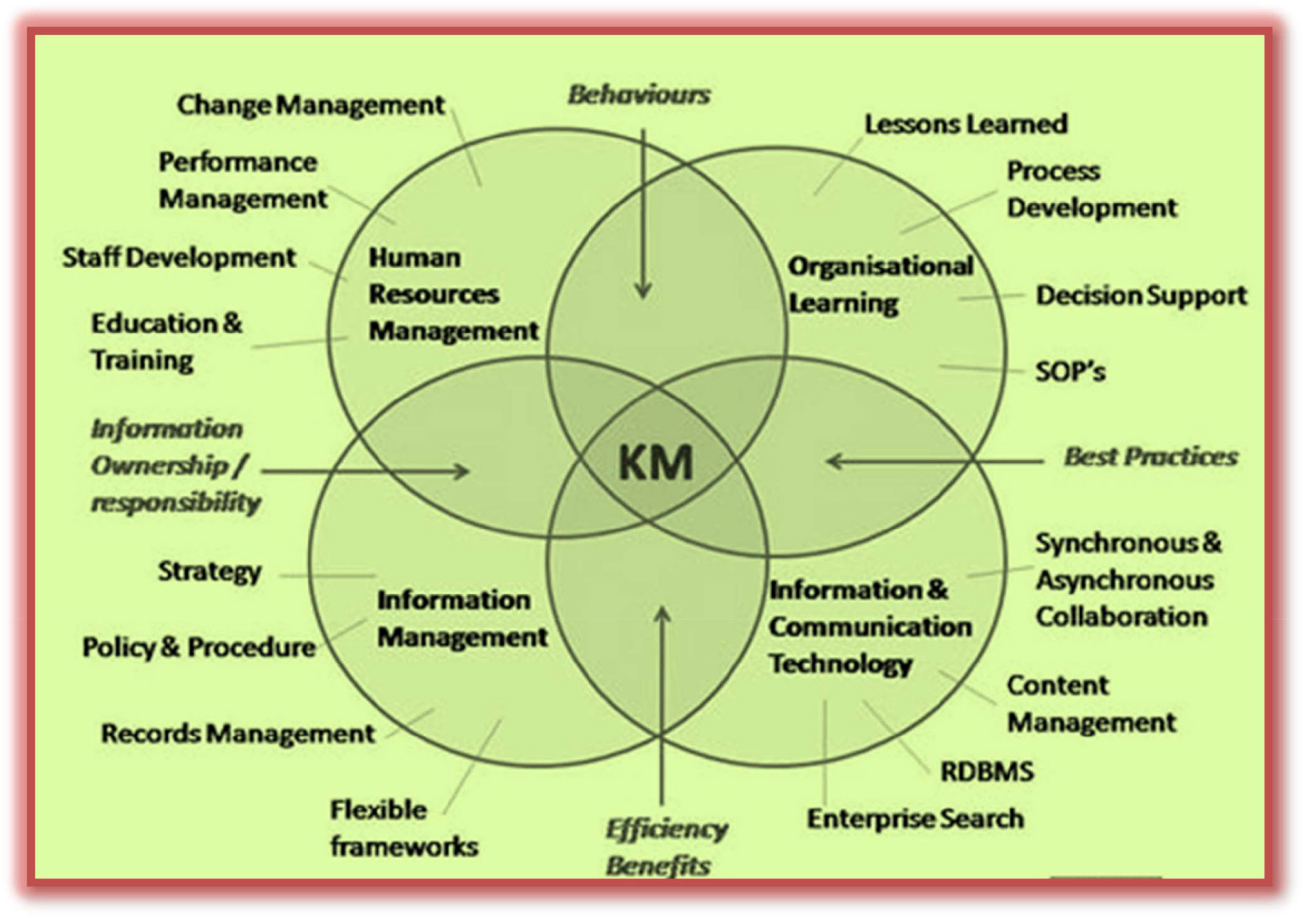
The link between HR and KM activities (source: authors, based on Garavan et al., 2001; Maier, 2002; Maier and Hädrich, 2006).

According to Gedam (2010), the HR function is vital to the development of a knowledge and competence management system to increase the competitiveness and sustainability of organizations. Moreover, human resources and information are two elements that can significantly impact an organization's overall effectiveness. Any of the two sources could be a source of competitive advantage, but corporate success typically needs superior management of both. Furthermore, the connection between these two is equally crucial. This is particularly feasible for the HR department, as they may leverage IT to develop new products and services and increase competitiveness. As discussed previously, the HR function encompasses the recruiting, selection, placement, assessment, and development of human resources, allowing for the automation of the HR function. Consequently, computerized employee record keeping, payroll administration and benefits administration, and transaction processing software were implemented. As the HRM concept has matured and is being implemented in Europe and North America, computer-based information systems have become a necessity. Indications from anecdotes point to a parallel evolution in Asian nations are also visible (Martinsons, 1997). Modern information technology provides complex data management, comprehensive, extensive, yet flexible reporting, decision support, and expert-level guidance. By emphasizing in-house HR difficulties and predicting future moves by competitors, contingency reporting can aid in preventing potential unanticipated events. For them, timely knowledge is crucial for ambiguous decisions (mainly involving expert judgment) that typically result in urgent impacts. The initial “wave” of KM research seemed hesitant about the need to “collect, codify, and distribute (usually in centrally governed computer systems) the firm's knowledge” (Gedam, 2010). Positive progress has been noticed over time, with a focus on people-oriented strategies in businesses as a means of managing knowledge (refer to Figure 5).

Similarly, competency management includes the logical examination, appraisal and assessment, visualization, development, and use of personnel competencies. In addition, competence management includes expertise locators, yellow and blue pages, and “skill management systems, sometimes known as people-finder systems” (Maier and Hädrich, 2006). The aforementioned skill management involves an information system that makes skill profiles readily available. Furthermore, it includes career and learning routes that are clearly outlined and determined for each employee and are regularly updated in conjunction with employees' skills profiles. The skill tree-named centralized skill ontology is detailed and classified. It provides a framework for all available, required, and desired abilities inside an organization. Training interventions are offered as well. These skill management systems often include information regarding employment status, projects, and training interventions through which workers developed, utilized, and enhanced their abilities. Additionally, the yellow and blue pages constitute an internal and external encyclopedia of organizational specialists. A profile of these professionals together with their contact information is provided in relation to a list of expertise areas, such as the article's keywords, for contacting the appropriate specialist. Details of specialists' skill levels and areas of competence can aid, for example, grouping individuals, hiring for projects, sorting, and customizing KMS content and functions.

According to Garavan et al. (2001), individual competencies are found on tacit knowledge. The tacit knowledge is acquired on the job through exposure to a certain circumstance or area. Tacit knowledge may be defined as the practical knowledge that people acquire within an organization. Furthermore, the tacit knowledge may derive from the organization's ideals and may be reflected in its socialization practices, rituals, and culture. The tacit knowledge accumulates through a process of collective learning. This results in the gradual customization of individual norms, behaviors, and attitudes to enhance person-organization compatibility. In reality, tacit knowledge exists in the minds of individuals. It is personal and unique to each individual, whereas explicit knowledge may be communicated and accessible. The fundamental disadvantage of tacit knowledge is that it is more difficult to explain in the proper language. As a result, workers with tacit knowledge amass higher negotiating power than others. However, it may be assumed that competencies are anchored in tacit knowledge. Hence, a person's competencies may be derived from his tacit knowledge. Consequently, tacit knowledge management is probably and frequently viewed as an alternative means of capturing human competencies (Venkitachalam and Busch, 2012).

On the other hand, HRM (i.e., HR department) represents an organizational subsystem in an institutional logic that plans, formulates, and executes economically acceptable employees' decisions. This is principally done to protect the availability and usefulness of personnel. HRM manages all relevant programs and functions required by an organization. This starts from hiring and selecting an employee and then leading toward his development and placement in the organization. Then by clarifying, for instance, personage behavior, enthusiasm, performance, and leadership (Maier, 2002) all persuade the management of knowledge in organizations. Furthermore, it is the human resource development function of HRM that has shaped most of the concepts of organizational learning (OL) and knowledge management (KM; refer to Figure 5).

Human resource management can facilitate recognizing the “critical knowledge base, knowledge barriers and gaps” required to describe a knowledge management strategy. Organizational learning and KM approaches have a tendency to apply a decentralized attitude to HRD by giving due importance to individuals and collectives. For instance, “collectives are work groups, teams as well as networks and communities in which members learn on the job, share knowledge and thus learn from each other” (Maier, 2002). In any case, for further centralized execution of KM strategies, meticulous and systematic planning of learning and development initiatives should be a prerequisite. This would also involve conventional HRM in an institutionalized sense. Thus, HRM contributes to a massive fraction of its job through organizational level KM initiative (Wiig, 1999).

Human resource management function may be better placed for instance, “for knowledge identification and mapping, to identify knowledge gaps and barriers, for general education and training programs and to foster an organizational culture supportive for KM and thus ensure the success of KM initiatives” (Maier and Hädrich, 2006).

### HRIS as knowledge and competence management system: Influencing strategic decision making

**Figure 6:**
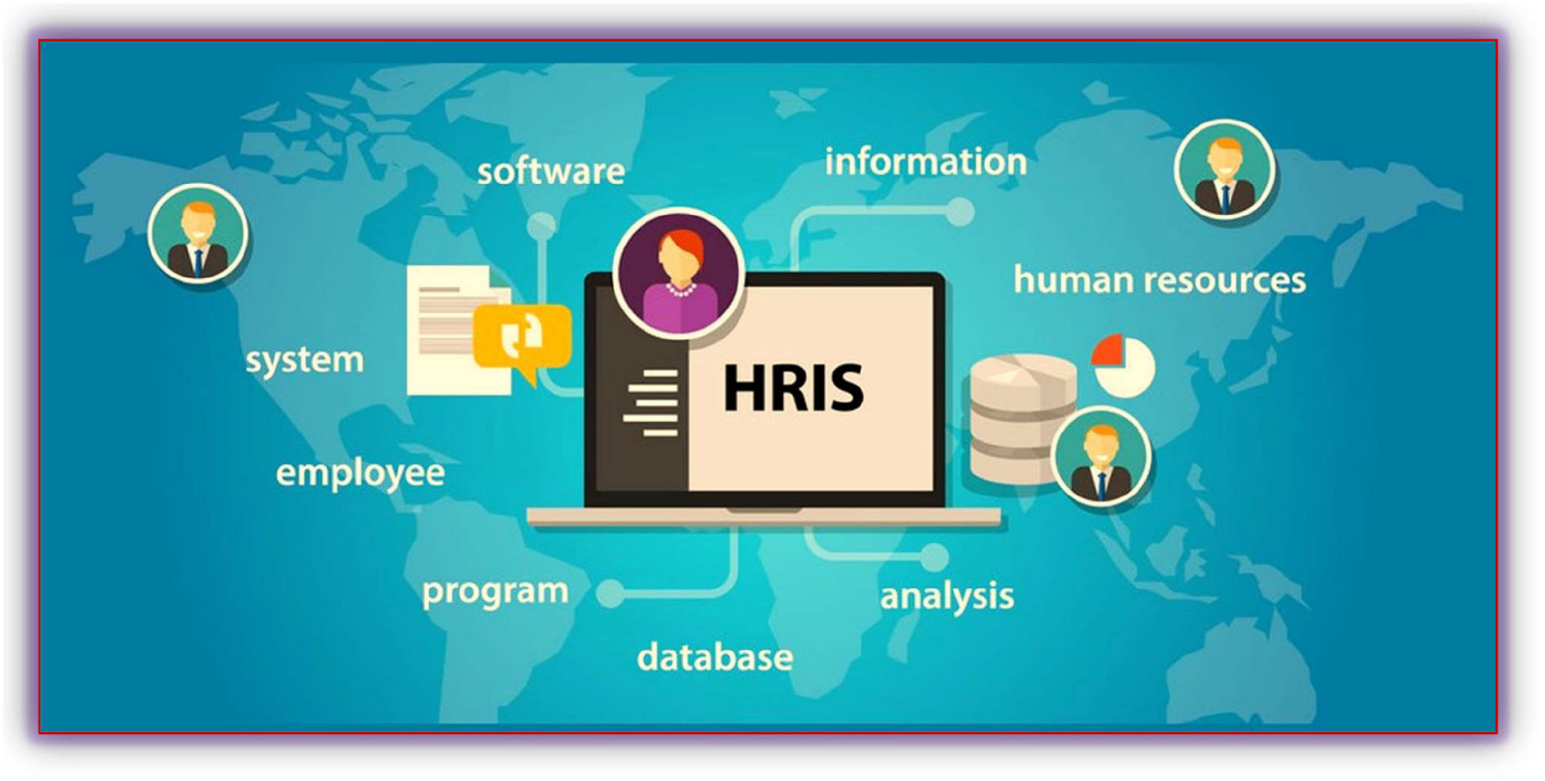
Components of HRIS (source: authors, based on Ball, 2001; Markova, 2012; Kazmi and Naaranoja, 2014).

Sveiby (2001) defines knowledge management in two ways: “management of information (knowledge = object which can be identified and dealt with by information systems) and management of people (knowledge = processes, a complex set of dynamic skills, which are constantly changing).” According to Chakraborty and Mansoor (2013), given that HRIS combines these two resources, i.e., people and information, proper adoption of the system can drive the organization to greater success and overall business performance. HRIS can be designed and customized in various ways, as per the knowledge and perception of the personnel involved (refer to Figure 6: components of HRIS). For instance, HRIS may be used simply as a computerized record-keeping system for automating HR routine processes; or it may be used for strategic purposes of managing human capital with decision support protocol and KMS (Desouza and Awazu, 2003; Tansley and Newell, 2007; Markova, 2012; Strohmeier and Kabst, 2012). In this regard, a customized and specifically designed application can fulfill the objectives of the organization (Markova, 2012; Kazmi and Naaranoja, 2014).

The purpose of HRIS is to provide employees with up-to-date, simplified information *via* intranet and HR-provided self-service to expand their knowledge. HRIS is an application capable of accumulating and disseminating knowledge (Ball, 2001; Markova, 2012). So, it is suggested that HRIS can synergistically integrate the two assets, i.e., people and information, and become the source of competitive advantage (Teo et al., 2007). In addition, HRIS influences organizational learning by facilitating the exchange of experiences between numerous users (Markova, 2012). However, developing the technical infrastructure for HRIS necessitates the use of information technologies. This includes particularly database and communication technologies to facilitate the development and maintenance of KM interventions and programs (Earl, 1996; Tansley and Newell, 2007; Strohmeier and Kabst, 2012). For instance, Infosys, which employs over 276,000 employees now, has implemented the concept of an internal knowledge market known as *K*-shop. On a website, workers publish their research papers, learning, and experience from various projects, case studies, and other items that serve as information sources. These documents are evaluated by specialists and made accessible to the public, while other employees may access them *via* a system. However, internal portals connected to HRIS provide access to information resources (Desouza and Awazu, 2003).

As a result, the fundamental idea of knowledge management is to utilize current ICTs as an enabling mechanism. It is a method for the implementation of organizational tools to build the system an organization employs to manage its knowledge (Dorasamy et al., 2013; Sarka and Ipsen, 2017). Therefore, it is recommended that HRIS software offers a dual advantage, i.e., it may serve as KMS as well as tools for managing HR routine operations such as payroll and benefits administration and personnel recordkeeping (Hendrickson, 2003; Teo et al., 2007). However, it is yet to be determined empirically to what degree the theoretical studies and hypotheses regarding HRIS's capabilities to function as KMS are accurate.

Human resource information system is believed to expedite the delivery of superior reports and analyses to higher-ups for informed decision-making. Tasks performed by HRIS for the convenience of determining strategic position in the market and making decisions are significant for achieving competitive advantage. HRIS functions include collecting, assembling, manipulating, analyzing, retrieving, and disseminating pertinent human resource information. Today, HRIS plays a crucial role in a variety of organizations. Even so, by the mid-1990's, the focus of HRIS had begun to shift as a result of its efficacy in strategic decision-making (Greer, 1995; Huselid, 1995; Kovach et al., 2002). Accordingly, the strategic applications of HRIS were classified as HR planning, recruiting and performance management, employee benefits, assessment and training requirements, compensation advice, and industrial relations (Hussain et al., 2007).

Human resource information system is comprised of interconnected modules that work together to offer the true picture of a company to make strategic decisions. The same is done by the human resource department being the backbone of every firm. HRIS's information is accurate and reliable, providing the foundation for enterprise-wide choices. It is a web-based application where data can be entered, tracked, and other pertinent tasks may be conducted with ease. It is commonly seen as an information service provided to businesses (Tannenbaum, 1990). HRIS can improve the efficacy of a variety of processes, whether it involves the accuracy of the information or the simplification and facilitation of the process through the use of technology. According to Lengnick-Hall and Moritz (2003), HRIS can initiate at three distinct levels: (1) distribution of information; (2) computerization of routine operations; and (3) bestowing a dynamic approach that transforms HRM into an organizational strategic partner rather than a routine administrative function. HRIS can improve operational effectiveness by expediting work completion, enhancing staff communication, and increasing accuracy and efficiency while reducing costs. “An HRIS can facilitate the development of strategic value by assisting in the formulation and implementation of internally consistent policies and practices that ensure human capital contributes to the achievement of company objectives” (Kassim et al., 2012).

The HRIS self-service accessed through the intranet supplies up-to-date and simplified information for grooming employees internally. Today, the intranet is utilized by bigger organizations for sentiment surveys, online appraisals, registrations for training, career management, and broadcasting employee-related business information. Similarly, HRIS software can also be integrated with email and desktop applications. Within the HRIS, word documents amalgamate and schedule through triggered actions to monitor activities. Furthermore, the documents can be produced, engagements reserved and confirmed through checking, and emails can be prepared and forwarded. The most recent and greatest HRIS takes account of time-clock systems, web GUI's, real-time systems and scanned data, and online analytical processing databases (OLAP). These OLAP permits express connections for online systems through which the data are inserted through the web. Therefore, while the information is stored, it is sent through an express connection to the HRIS. Technical infrastructure building entails the use of IT, especially database and communication technologies, to smooth the progress and sustain the KM activities. The fundamental idea of KM is to apply contemporary ICTs as an enabler. It is a mechanism for organizations to put their instruments into practice to advance the system in which an organization manages knowledge.

Thus, such advanced HRIS applications as discussed above have dual advantages, i.e., they can act as knowledge and competence management systems as well as a tool for managing HR operational activities such as payroll and benefits administration and personnel record management applications.

## Barriers to implementation of HRIS as KMS

Even in bigger enterprises, HRIS is the least chosen for strategic goals; instead, it is employed for mundane operational activities, such as record keeping and payroll services solely (Altarawneh and Al-Shqairat, 2010; Spero et al., 2011; Bamel et al., 2014; Qaisar et al., 2018). This is bad since the system is not being utilized for what was intended when it was created and funded. It may be seen as a sunk cost. The key factors listed below may be viewed as obstacles even after HRIS installation.

### Dependence of HRIS on the IT department

The reasons communicated by some of the researchers are the lack of staff, the lack of information technology (IT) support, the need to work with other departments, inadequate knowledge for the implementation of the system, and the lack of expertise in IT (Ngai and Wat, 2006; Nusair and Parsa, 2007; Troshani et al., 2011; Bamel et al., 2014). These reasons are more leaning toward information technology/system's functional failure and in-capabilities.

Since HRIS characterizes the integration between HRM and IT, therefore, these systems may be dependent on centralized hardware resources. Still, some information system specialists within the HR department support HR in managing and maintaining these resources as well as applications (Hendrickson, 2003). Several researchers present that organizations that are IT sophisticated (i.e., have a properly-recognized IT department as well as IT assets, such as IT capabilities and knowledge) may tend to adopt IT innovations more quickly (Bassellier et al., 2003; Tsai et al., 2010; Roztocki and Weistroffer, 2011). The availability of relevant IT skills in employees for technological innovations (Ulrich et al., 1995; Zhu et al., 2003) does play a critical role in the HRIS adoption process. Thus, this corresponding aspect has been recognized in many reports as a fundamental component of IT applications' implementation (Brynjolfsson and Hitt, 2000; Black and Lynch, 2001; Al-Dmour et al., 2016). For this study, IT skills are defined “by the quantity of employees who work exclusively on tasks related to IT activities and those who have HRIS expertise” (Al-Dmour et al., 2016). Furthermore, to assess the number of staff deputed to the HRIS project, their role is also counted for the successful implementation and usage of HRIS.

### HR, HRIS, and use of technology

The second dimension of research showed that being a non-technical department, HR has been dependent on IT/IS departments. HRIS has been limited and difficult for normal HR professionals to use, modify, and maintain. In general, HR professionals do not have computer expertise, even some companies are outsourcing the tasks related to the use of IT (Ulrich et al., 2008; Delorme and Arcand, 2010; Roztocki and Weistroffer, 2011; Troshani et al., 2011; Qaisar et al., 2018). Society for Human Resource Management (2003) in its study depicted the result as 28% proficiency against a measure “ability to leverage technology for HR practices to deliver value,” i.e., a competency entitled “HR technology” (Al-Dmour et al., 2016), as compared to the scores varying from 47 to 73% for the other critical competencies (Society for Human Resource Management, 2003). This dimension refers to the HR department's lack of HRIS expertise and other technological innovations.

The increasing deployment of HRIS and other technological products in organizations globally elicits the need to seek the latest competencies for HR personnel (Gardner et al., 2003; Delorme and Arcand, 2010; Unal and Mete, 2012; Qaisar et al., 2018). These competencies are critical since HRIS makes the use of the internet, intranet, communication networks, databases, data mining tools, etc. (Olughor, 2016). So, HR workers need to work hard and change with the times, especially in the last 20 years (Haines and Petit, 1997; Mayfield et al., 2003; Delorme and Arcand, 2010).

This explosive technological change has created challenges for HR to speedily get up and transform traditional processes into online and technology-oriented processes. Furthermore, being the custodian of an organization's human capital, HR is under additional pressure to manage knowledge and human capital (Hendrickson, 2003; Hempel, 2004; Teo et al., 2007). HRIS is the tool that can resolve this issue of managing knowledge and human capital through organizational and individual learning (Mayfield et al., 2003; Teo et al., 2007). However, a critical component in an organization's continued success and excellence in HRIS is the abundance of expert HRIS personnel (Unal and Mete, 2012). Users' blankness toward the system's functionalities and modules is the most important faltering block in HRIS usage and implementation (Teo et al., 2007). Therefore, expertise is a critical factor in the underutilization of new technologies. HRIS expertise refers to “employees' knowledge of and technical competence in HRIS” (Al-Dmour et al., 2016). HRIS personnel should be multidisciplinary and should have practical knowledge of information systems (IS) working along with HR functions (Elliot and Tevavichulada, 1999; Roberts, 1999). The slow pace at which information technology has been functional in HR departments is attributed to being deficient in HRIS knowledge and skills as well as the complex nature of HR systems for non-technical professionals (Teo et al., 2007; Al-Dmour et al., 2016). In general, IT/IS departments maintain HRIS wherever it has been adopted, since HRIS has proven to be difficult for normal HR professionals (Dunivan, 1991; Delorme and Arcand, 2010). But HR professionals need to know a lot about relational databases, data mining, communication media and applications, simulation modeling, expert system design, and the difference between managing knowledge workers and managing manual workers (Hempel, 2004; Haines and Lafleur, 2008).

### Top management support and commitment

Another stream of research presents the barriers; for instance, the lack of management support; HRIS not being perceived as advantageous; difficulty in changing organizational culture; fear of new or changing ways (Ngai and Wat, 2006; Nusair and Parsa, 2007; Troshani et al., 2011; Bamel et al., 2014). These are based on top management support, commitment, and attitude toward the adoption of change through the change in organizational culture. These activities need a high level of interest from top management since top management has to decide regarding the adaptation and implementation of HRIS applications. The support from higher-ups would ultimately decrease the resistance at lower levels (Troshani et al., 2011; Chakraborty and Mansoor, 2013). Many researchers (Razali and Vrontis, 2010; Troshani et al., 2011; Bamel et al., 2014; Alam et al., 2016; Khan et al., 2017) have found that top management support can help overcome potential problems after HRIS is implemented.

In fact, the decisions regarding adaptation and implementation of HRIS, the cost to incur on the application, the extent to which the organization will customize the application, and allowing HR/HRIS to interfere in strategic decision-making are all top management decisions. These are only possible through top management's support (Wiblen et al., 2010; Schalk et al., 2012). The authors Ngai and Wat (2006) argue that the implementation of HRIS is also dependent on the size of the company. Large companies have established facilities such as extranets; intranets, and so on, whereas smaller companies lack these resources. In fact, it is the top management that has to provide the budget and other resources for the implementation of HRIS. But, due to the lower understanding of the potential benefits of HRIS, management is not willing to allocate the required resources. Thus, HR professionals need to carry out promotional activities and present action plans in front of top management. HR has to demonstrate the real advantages and benefits of the implementation of HRIS (Ngai and Wat, 2006). In this way, HR professionals may be able to gain top management's support and commitment and get the required budget/resources for the implementation of HRIS.

## Conclusion and future research

Knowledge and competency management has long been a top priority, but a formidable challenge for organizations. In today's age of globalization and technology, the competition has become fierce with regard to product quality, price, services, delivery, and so on. Therefore, businesses need to gain knowledge and skills more quickly than competitors to stand out and thrive (Garavan et al., 2002). Human capital, with the necessary knowledge and skills, is the primary mechanism by which any type of business may acquire a competitive advantage and influence its overall performance (Memon et al., 2018).

The knowledge retained by an organization's employees may serve as its foundation. Any KM solution will benefit greatly by having access to the archive of the company's major decisions and events. Data can be gathered, then processed, analyzed, then retrieved, and shared with others *via* the HRIS. HRIS is an alternative to KMS. HRIS may be used in two ways that give businesses a leg up on the competition: (1) as an administrative tool for handling the day-to-day tasks associated with managing the human resources department, and (2) as a tool for keeping track of employees' skills and knowledge. To get the most out of HRIS, however, businesses need to use customized and specialized software (Gedam, 2010). Information and communication technology (ICT) advancements in the form of an HRIS have made it possible to build an environment conducive to the production of new knowledge and the building of new networks. In addition to a more rapid response time, HRIS also provides more reliable and consistent data. HRIS that fosters organizational competency *via* efficient KM should, ideally, mirror these characteristics and provide clarity in their design through a framework (for instance; the framework in Figure 4).

Human resource departments may play a crucial role in restoring their lost value if they acquire the essential HRIS skills and persuade the top management. Specifically, in small organizations, HR will need to put extra effort to obtain the support of senior management by demonstrating the potential benefits and concrete results of deploying HRIS. As a result of these actions, HR will be able to use HRIS in strategic planning.

The said research was limited to describing, analyzing, and theoretical review of the topic being a structured review article. However, future researchers may carry out the empirical analysis of the organizations having implemented HRIS as KMS. Furthermore, the technical configuration, customized components, technology, and expert systems used in HRIS implementation may be investigated through a detailed analysis and case study approach.

## Data availability statement

The original contributions presented in the study are included in the article/supplementary material, further inquiries can be directed to the corresponding author.

## Author contributions

All of the authors contributed to conceptualization, formal analysis, investigation, methodology, and writing and editing the original draft. All authors contributed to the article and approved the submitted version.

## Conflict of interest

The authors declare that the research was conducted in the absence of any commercial or financial relationships that could be construed as a potential conflict of interest.

## Publisher's note

All claims expressed in this article are solely those of the authors and do not necessarily represent those of their affiliated organizations, or those of the publisher, the editors and the reviewers. Any product that may be evaluated in this article, or claim that may be made by its manufacturer, is not guaranteed or endorsed by the publisher.
